# Retraction Note: Neonatal congenital mesoblastic nephroma that caused respiratory oncologic emergency early after birth: a case report

**DOI:** 10.1186/s12887-022-03817-x

**Published:** 2022-12-29

**Authors:** Hirotaka Kato, Yasuyuki Mitani, Taro Goda, Hiroki Yamaue

**Affiliations:** Second Department of Surgery, Wakayama Medical University Hospital, 811-1 Kimiidera Wakayama, Wakayama, 641-8509 Japan

**Retraction Note: BMC Pediatrics 22, 139 (2022)**

**https://doi.org/10.1186/s12887-022-03210-8**

The authors have retracted this paper because it has been previously published by an overlapping author group [1]. The authors stated that they did not obtain the permission to republish the article in English. All authors agree to this retraction.
